# Glycaemia and hand grip strength in aging people: Guangzhou biobank cohort study

**DOI:** 10.1186/s12877-020-01808-0

**Published:** 2020-10-12

**Authors:** Xue Liang, Chao Qiang Jiang, Wei Sen Zhang, Feng Zhu, Ya Li Jin, Kar Keung Cheng, Tai Hing Lam, Lin Xu

**Affiliations:** 1 School of Public Health, Sun Yat-sen University, Guangzhou, China; 2 Guangzhou No.12 Hospital, Guangzhou, 510620 China; 3 Institute of Applied Health Research, University of Birmingham, Birmingham, UK; 4 School of Public Health, the University of Hong Kong, Hong Kong, China

**Keywords:** Glycaemia, Prediabetes, Normoglycaemia, Grip strength

## Abstract

**Background:**

There is a link between hyperglycemia and mechanical functions of muscle. However, existing evidence of the association between hyperglycemia and weaker muscle strength is limited and inconsistent. We examined whether glycemic status was associated with relative grip strength (RGS) in older Chinese.

**Methods:**

In 2008–2012, 9180 participants (2516 men and 6664 women) from the Guangzhou Biobank Cohort Study had fasting and 2-h post-load glucose measured. Glycemic status was categorized as normoglycaemia, prediabetes (i.e., impaired fasting glucose and/or impaired glucose tolerance) and diabetes. RGS was assessed using a Jamar Hydraulic Hand Dynamometer divided by body mass index. General linear model was used to assess the association of glycemic status with RGS.

**Results:**

After adjusting for age, smoking status, alcohol use, physical activity, health status, body fat percentage and waist circumference, in men, hyperglycemia was associated with a lower RGS, with the RGS being 1.38 (95% confidence interval (CI) = 1.34, 1.42) in normoglycaemia, 1.35 (95% CI = 1.30, 1.39) in prediabetes, 1.33 (95% CI = 1.29, 1.38) in newly diagnosed diabetes and 1.32 (95% CI = 1.27, 1.37) in known diabetes (*P* for trend < 0.001). The association of glycemic status with RGS was non-significant in women. Among the normoglycaemic group, no association was found between fasting glucose and RGS in men, whereas a significantly inverse association was found in women, with adjusted β for RGS per mmol/l increase in fasting glucose being − 0.05 to − 0.04 (*P* values from 0.002 to 0.03).

**Conclusions:**

Higher fasting glucose was associated with reduced grip strength in a dose-response manner, and the association was significant even in women with normoglycaemia. Our findings suggest that lowering glucose across the whole range might be important in preserving muscle strength, especially in aging women.

## Background

Low muscle strength is an indicator for frailty [1] which is more prevalent in people with diabetes than those without [2], and predicts higher risks of disability, falls [3] and mortality [4]. Grip strength has been shown to be a good proxy for general muscle strength [5, 6], especially in older people [5]. Identifying modifiable factors associated with grip strength may enable effective primary and secondary prevention strategies in preserving ageing-related muscle loss.

Diabetes mellitus (DM) is a major chronic disease with severe complications. The number of DM patients has reached 463 million globally in 2019 and is estimated to increase to 700 million in 2045 [7]. China has the largest number of DM patients in the world, with more than one tenth of Chinese adults having DM in the recent decade [8, 9]. Diabetes is associated with poorer physical performance and disability in older people [10, 11]. However, there are limited data describing the risk of poor physical performance from glycemic measures, and increased risk may begin at levels below the current diagnostic criteria for prediabetes or diabetes. To our knowledge, there were only three papers with inconsistent results. One showed that type 2 diabetes mellitus (T2DM) was associated with lower grip strength in men and the association was less pronounced but still significant in women [12]. Another reported a positive association between fasting glucose and grip strength in men but not in women, and no association between 2-h post-load glucose (2hPG) and grip strength in men or women [13]. The third found that muscle strength was significantly lower in the highest versus lowest quartile of hemoglobin A_1c_ but sex differences or fasting glucose and 2hPG were not reported [14]. We therefore examined the association between glycemic status and grip strength using a large sample of middle-aged to older people from the Guangzhou Biobank Cohort Study (GBCS).

## Methods

### Study sample

All participants were from GBCS, which is an on-going three-way collaborative project of the Guangzhou 12th Hospital and the Universities of Hong Kong, China and Birmingham, United Kingdom. Details of GBCS have been reported elsewhere [15]. Briefly, all participants were recruited from the Guangzhou Health and Happiness Association for the Respectable Elders (GHHARE), a large social and welfare organization. Guangzhou permanent residents aged 50+ years were eligible to participate, with a monthly membership fee of 4 RMB (about 0.57 USD). The GHHARE included about 7% of residents in this age group, with branches over all districts of Guangzhou. The study was approved by the Guangzhou Medical Ethics Committee of the Chinese Medical Association. All participants provided written informed consent before participation.

### Measures

In this paper, we used data from participants who returned for the second examination during March 2008 to December 2012. Age range of the participants was from 53 to 98 years, with the mean age being 68 and 65 years for men and women, respectively. Within age-group, it has been shown that participants had similar levels of diabetes and hypertension to the nationally representative samples of urban Chinese [15–17]. In 9195 participants, after excluding those with missing information (*n* = 15), 9180 participants were included in the analyses. Face-to-face interviews [15] were conducted to collect baseline information by trained nurses including demographic characteristics, lifestyle, and personal and family medical history. Body mass index (BMI) was calculated using measured weight and height as kilograms divided by meters squared. Education was self-reported and classified into three groups: primary or below (0-6 years), middle school (7–12 years) and college or above (≥13 years). Smoking status was defined as having smoked at least one cigarette per day or 7 cigarettes per week for at least half a year, and classified into three groups: never (those did not smoke during their life time), former (used to smoke but not smoking currently) and current (answering ‘yes’ to the question: ‘do you smoke cigarettes now?’). Alcohol use was classified into three groups: never (those never consumed any alcoholic beverage during their life), former (those stopped drinking for more than one year) and current (those drunk any alcoholic beverage in the past 12 months). Physical activity was assessed by a validated Chinese version of the International Physical Activity Questionnaire [18] and classified into three groups: active (vigorous activity ≥3 days a week achieving at least 1500 metabolic equivalent values (MET) or moderate activity ≥3000 METs daily), minimally active (vigorous activity ≥3 days a week achieving 480 METs or any combination of walking, moderate or vigorous activities ≥5 days a week achieving 600 METs) and inactive (those did not meet the criteria for active or minimally active). As age [19], education [20, 21], lifestyle factors (smoking status, alcohol use [22] and physical activity [23]), health status and anthropometric parameters (body fat percentage [24], waist circumference [25] and BMI [26]) may be associated with both glycaemia and grip strength, these factors were considered as potential confounders and adjusted in the multivariable models.

### Exposures

**Fig. 1:**
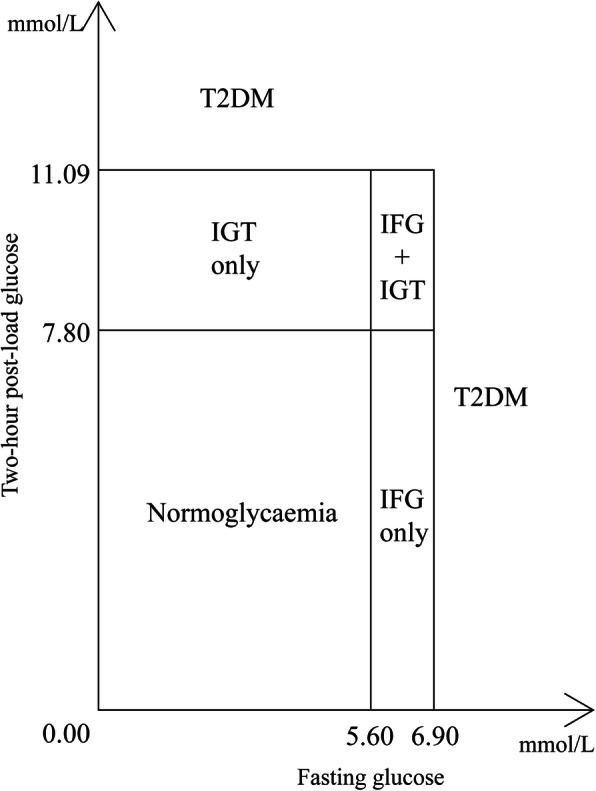
Classification of glycemic status by fasting glucose and two-hour post-load glucose levels. IFG only, impaired fasting glucose only, i.e., fasting level of 5.6–6.9 mmol/l and 2hPG < 7.8 mmol/l; IGT only, impaired glucose tolerance only, i.e., 2hPG level of 7.8–11.09 mmol/l and fasting glucose < 5.6 mmol/l, IFG + IGT, the presence of both impaired fasting glucose and impaired glucose tolerance, T2DM type 2 diabetes mellitus, i.e., fasting glucose ≥7.0 mmol/l or 2hPG ≥11.1 mmol/l; Known T2DM a history of self-reported physician-diagnosed diabetes or the use of glucose-lowering drugs regularly

All participants were required to fast for at least 10 h from the night before blood taking in the morning. Fasting glucose were measured by Shimadzu CL-8000 Clinical Chemistry Analyzer (Shimadzu, Kyoto, Japan). Two-hour post-load glucose (2hPG) was measured after 75-g oral glucose administration in all participants except for those with self-reported physician diagnosis of diabetes or with glucose-lowering treatment. Participants were categorized into six groups, including (1) normoglycaemia, (2) impaired fasting glucose (IFG) only, (3) impaired glucose tolerance (IGT) only, (4) IFG and IGT, (5) newly diagnosed T2DM and (6) known T2DM [27]. Although we found higher fasting glucose was associated with higher 2hPG, of those who had prediabetes, those with IFG/IGT only had only one glycemic impairment indicating the other glycemic function was fairly normal. IFG + IGT were those had higher glycaemia in both fasting glucose and 2hPG, which had higher risk for T2DM than IFG/IGT only [28]. The six exposure groups were classified from normoglycaemia to prediabetes then to diabetes (Fig. 1). Normoglycaemia was defined by both fasting glucose < 5.6 mmol/l and 2hPG < 7.8 mmol/l. According to American Diabetes Association [28], IFG only was defined by a fasting glucose level of 5.6–6.9 mmol/l and 2hPG < 7.8 mmol/l. IGT only was defined by a 2hPG level of 7.8–11.09 mmol/l and fasting glucose < 5.6 mmol/l. IFG and IGT (i.e., IFG + IGT) was defined by the presence of both impaired fasting glucose and impaired glucose tolerance (i.e., a fasting level of 5.6–6.9 mmol/l and a 2hPG level of 7.8–11.09 mmol/l). Prediabetes was defined as IFG and/or IGT. Newly diagnosed T2DM was defined by fasting glucose ≥7.0 mmol/l or/and 2hPG ≥11.1 mmol/l and without known T2DM. Known T2DM was defined by a history of self-reported physician-diagnosed diabetes or glucose-lowering treatment.

### Outcomes

Grip strength was assessed using a Jamar Hydraulic Hand Dynamometer in a standing position. Grip strength of each hand was tested two times and the average value was calculated, expressed as kilograms. The maximal reading of the average grip strength in right and left hands was used as the absolute grip strength (AGS). Measurements of grip strength using Jamar dynamometer showed good to excellent test-retest reproducibility (r > 0.80) [29] and excellent (*r* = 0.98) inter-rater reliability [30]. Maximum relative grip strength (RGS _max_) was calculated by AGS divided by BMI. Average relative grip strength (RGS _mean_) was calculated by the average grip strength in both hands divided by BMI. Relative grip strength in left- or right- hand (RGS _left_ / RGS _right_) was calculated by the average grip strength in left- or right- hand divided by BMI [31, 32].

### Statistical analysis

Pearson χ^2^ test and one-way analysis of variance (ANOVA) were used to compare categorical and continuous variables respectively. The Bonferroni’s test was used to control the family-wise error rate. General linear models were used to assess the association of glycemic status and quartiles of fasting glucose in normoglycaemic group with grip strength, giving adjusted regression coefficient (β) and 95% confidence intervals (CIs). We also tested for interaction between sex and glycaemia groups. As significant interactions were observed on the associations with RGS _mean_ and RGS _right_ (P for sex interaction from 0.03 to 0.92), all analyses were done on total participants and by sex. Matrix diagram of the measures of grip strength were plotted, with the RGS _max_ showing the highest correlation coefficient than other measures of grip strength (Supplementary Fig. 1). Thus, we selected RGS _max_ as a proxy to investigate the association of fasting glucose and 2hPG with RGS _max_. Potential confounders were classified as in Table 1. We compared models using different categorizing methods for RGS _max_ including tertiles, quartiles and quintiles in terms of model fitness and found that these three methods showed similar fitness as indicated by the values of Akaike Information Criterion. Hence, to enable comparison with previous studies [21, 33], we categorized RGS _max_ into quintiles. Participants’ characteristics by sex-specific quintiles of RGS _max_ are shown in the Supplementary Table 1. Statistical analyses were done using Stata version 16.0 (STATA Corp LP, College Station, Texas, USA).

**Table 1:** Characteristics by diabetes status in 2516 men and 6664 women from the Guangzhou Biobank Cohort Study

	Normoglycaemia	IFG only	IGT only	IFG + IGT	T2DM	Known T2DM	*P* value
Men							
Number	1271	224	363	150	209	299	
Fasting glucose, mmol/l, mean (SD)	4.93 (0.42)	5.93 (0.28)	5.08 (0.44)	6.01 (0.30)	7.09 (2.31)	7.70 (3.15)	< 0.001
Post-load glucose, mmol/l, mean (SD)	6.03 (1.26)	6.47 (1.37)	8.90 (0.84)	9.17 (0.90)	14.42 (4.32)	9.25 (4.33)	< 0.001
Age, years, mean (SD)	67.38 (6.68)	67.28 (6.71)	69.38 (6.49)	68.31 (6.68)	69.18 (5.66)	68.88 (6.37)	< 0.001
Education, %							
Primary or below	27.87	27.68	25.34	24.00	30.62	25.42	0.11
Middle school	57.17	52.68	57.30	58.67	52.15	51.51	
College or above	14.96	19.64	17.36	17.33	17.22	23.08	
Smoking status, %							
Never	41.67	43.30	47.66	48.67	47.12	46.15	< 0.001
Former	24.86	29.02	29.20	30.00	30.29	31.77	
Current	33.46	27.68	23.14	21.33	22.60	22.07	
Alcohol use, %							
Never	29.43	34.38	28.93	32.67	35.89	37.12	0.003
Former	0.63	2.68	0.00	1.33	0.48	0.67	
Current	69.94	62.95	71.07	66.00	63.64	62.21	
Physical activity, %							
Inactive	2.28	1.34	1.38	0.67	2.87	1.34	0.24
Minimally active	27.14	25.00	30.30	24.67	34.45	28.76	
Active	70.57	73.66	68.32	74.67	62.68	69.90	
Poor health, %	60.82	58.48	56.98	59.31	61.54	64.43	0.49
Body fat, %, mean (SD)	21.04 (6.00)	23.35 (5.26)	22.47 (6.19)	24.47 (5.86)	24.47 (5.57)	23.01 (5.69)	< 0.001
Waist circumference, cm, mean (SD)	82.30 (8.60)	86.35 (8.95)	85.18 (9.13)	88.07 (8.82)	88.17 (9.09)	87.20 (8.66)	< 0.001
BMI, kg/m^2^, mean (SD)	22.86 (3.08)	23.97 (3.32)	23.83 (3.46)	25.03 (4.34)	24.66 (3.20)	24.44 (3.47)	< 0.001
Women							
Number	3395	487	993	391	558	840	
Fasting glucose, mmol/l, mean (SD)	4.94 (0.36)	5.90 (0.27)	5.10 (0.36)	6.00 (0.32)	6.90 (2.14)	7.69 (2.85)	< 0.001
Post-load glucose, mmol/l, mean (SD)	6.14 (1.03)	6.67 (1.18)	8.91 (0.83)	9.19 (0.94)	14.28 (4.11)	10.98 (5.06)	< 0.001
Age, years, mean (SD)	63.70 (6.84)	63.81 (7.10)	65.80 (6.51)	66.17 (6.74)	66.25 (6.61)	66.64 (6.41)	< 0.001
Education, %							
Primary or below	38.47	43.33	46.83	47.83	53.23	51.90	< 0.001
Middle school	54.46	52.77	46.02	44.76	40.32	41.07	
College or above	7.07	3.90	7.15	7.42	6.45	7.02	
Smoking status, %							
Never	97.48	97.32	97.77	98.19	97.84	97.12	0.35
Former	0.98	1.44	0.81	1.29	0.90	1.92	
Current	1.54	1.24	1.42	0.52	1.26	0.96	
Alcohol use, %							
Never	45.71	51.85	46.27	47.06	49.01	57.23	< 0.001
Former	0.27	0.00	0.30	0.00	0.72	0.36	
Current	54.03	48.15	53.43	52.94	50.27	42.41	
Physical activity, %							
Inactive	2.56	0.62	2.62	3.58	2.69	2.62	0.002
Minimally active	21.06	20.33	23.77	23.53	24.37	27.14	
Active	76.38	79.06	73.62	72.89	72.94	70.24	
Poor health, %	64.08	60.46	64.34	63.73	63.47	73.56	< 0.001
Body fat, %, mean (SD)	31.54 (6.74)	34.46 (6.74)	33.14 (6.71)	36.09 (6.56)	36.26 (6.66)	33.47 (6.69)	< 0.001
Waist circumference, cm, mean (SD)	79.69 (8.40)	82.99 (8.71)	82.17 (8.57)	84.95 (8.68)	85.67 (8.95)	83.90 (9.27)	< 0.001
BMI, kg/m^2^, mean (SD)	23.30 (3.50)	24.47 (3.47)	24.19 (3.36)	25.19 (3.23)	25.53 (3.72)	24.48 (3.58)	< 0.001

Post-load glucose, 2 h oral glucose tolerance test; *BMI* body mass index, *SD* standard deviation

## Results

Table 1 shows that in 2516 men and 6664 women, those with T2DM, versus normoglycaemia, were older and had higher body fat percentage, waist circumference, BMI and lower percentage of current alcohol use (*P* from < 0.001 to 0.003). There were also significant differences in physical activity, education and health status in women and smoking status in men with poorer status in T2DM group (*P* from < 0.001 to 0.002).

Table 2 shows that, after adjusting for age, education, smoking status, alcohol use, physical activity, body fat percentage and waist circumference, in men, women, and the total participants, RGS _max_ declined from normoglycaemia to prediabetes (from IFG only, to IGT only, then to IFG + IGT), then to known/newly diagnosed T2DM groups (*P* for trend = 0.02, 0.03 and 0.005, respectively). The associations of glycemic stages with grip strength were different in men and women in terms of RGS _mean_, RGS _left_, RGS _right_ and AGS. Although the associations of glycemic status with these measures of grip strength (i.e., RGS _mean_, RGS _left_, RGS _right_ and AGS) were not statistically significant in women (*P* for trend from 0.07 to 0.17), significant inverse associations were observed in men (*P* for trend from 0.01 to 0.03) as well as total participants (*P* for trend from 0.005 to 0.03).

**Table 2:** Grip strength by different diabetes status in 2516 men and 6664 women

	Normoglycaemia	IFG only	IGT only	IFG + IGT	T2DM	Known T2DM	*P* for trend^†^
Men							
Number of subjects	1271	224	363	150	209	299	
Relative grip strength _max_^†^	1.38 (1.34, 1.42)	1.37 (1.33, 1.40)	1.36 (1.32, 1.39)	1.35 (1.30, 1.39)	1.33 (1.29, 1.38)	1.32 (1.27, 1.37)	0.02
Relative grip strength _mean_^†^	1.30 (1.27, 1.34)	1.29 (1.26, 1.33)	1.28 (1.25, 1.31)	1.27 (1.24, 1.31)	1.26 (1.22, 1.30)	1.25 (1.21, 1.29)	0.01
Relative grip strength _left_^†^	1.30 (1.26, 1.33)	1.28 (1.25, 1.32)	1.27 (1.23, 1.31)	1.26 (1.22, 1.30)	1.25 (1.21, 1.29)	1.24 (1.19, 1.29)	0.01
Relative grip strength _right_^†^	1.31 (1.27, 1.35)	1.30 (1.27, 1.33)	1.29 (1.26, 1.33)	1.28 (1.25, 1.32)	1.27 (1.23, 1.31)	1.26 (1.22, 1.31)	0.01
Absolute grip strength, kg^††^	32.76 (31.88, 33.65)	32.52 (31.68, 33.36)	32.27 (31.43, 33.12)	32.03 (31.14, 32.92)	31.79 (30.80, 32.77)	31.54 (30.44, 32.64)	0.03
Women							
Number of subjects	3395	487	993	391	558	840	
Relative grip strength _max_^†^	0.91 (0.89, 0.92)	0.90 (0.89, 0.92)	0.90 (0.89, 0.91)	0.90 (0.88, 0.91)	0.89 (0.87, 0.91)	0.88 (0.86, 0.90)	0.03
Relative grip strength _mean_^†^	0.86 (0.84, 0.87)	0.85 (0.84, 0.87)	0.85 (0.84, 0.86)	0.85 (0.84, 0.86)	0.85 (0.83, 0.86)	0.84 (0.83, 0.86)	0.17
Relative grip strength _left_^†^	0.85 (0.84, 0.86)	0.85 (0.83, 0.86)	0.84 (0.83, 0.86)	0.84 (0.83, 0.85)	0.84 (0.82, 0.85)	0.83 (0.81, 0.85)	0.15
Relative grip strength _right_^†^	0.86 (0.85, 0.88)	0.86 (0.85, 0.87)	0.86 (0.85, 0.87)	0.86 (0.84, 0.87)	0.85 (0.84, 0.87)	0.85 (0.83, 0.87)	0.15
Absolute grip strength, kg^††^	20.82 (20.49, 21.14)	20.72 (20.43, 20.01)	20.62 (20.33, 20.91)	20.52 (20.20, 20.85)	20.42 (20.04, 20.81)	20.33 (19.87, 20.78)	0.07
Total							
Number of subjects	4666	711	1356	541	767	1139	
Relative grip strength _max_^$^	1.02 (1.01, 1.03)	1.01 (1.00, 1.02)	1.00 (1.00, 1.01)	1.00 (0.99, 1.01)	0.99 (0.98, 1.00)	0.99 (0.97, 1.00)	0.005
Relative grip strength _mean_^$^	0.96 (0.95, 0.97)	0.95 (0.95, 0.96)	0.95 (0.94, 0.96)	0.94 (0.94, 0.95)	0.94 (0.93, 0.95)	0.94 (0.92, 0.95)	0.03
Relative grip strength _left_^$^	0.95 (0.94, 0.96)	0.94 (0.94, 0.95)	0.94 (0.93, 0.94)	0.93 (0.92, 0.94)	0.93 (0.91, 0.94)	0.92 (0.91, 0.94)	0.02
Relative grip strength _right_^$^	0.97 (0.96, 0.98)	0.97 (0.96, 0.97)	0.96 (0.95, 0.97)	0.96 (0.95, 0.97)	0.95 (0.94, 0.96)	0.95 (0.93, 0.96)	0.02
Absolute grip strength, kg^$$^	23.79 (23.58, 24.06)	23.67 (23.50, 23.84)	23.54 (23.38, 23.71)	23.42 (23.21, 23.63)	23.30 (23.01, 23.58)	23.17 (22.81, 23.53)	0.008

Results were shown as mean (95% confidence interval), except for numbers

^†:^ Adjusted for age, education, smoking status, alcohol use, physical activity, body fat percentage and waist circumference

^††:^ Adjusted for age, education, smoking status, alcohol use, physical activity, body fat percentage and waist circumference and body mass index (BMI)

^$:^ Adjusted for age, sex, education, smoking status, alcohol use, physical activity, body fat percentage and waist circumference

^$$:^ Adjusted for age, sex, education, smoking status, alcohol use, physical activity, body fat percentage and waist circumference and BMI

*IFG* impaired fasting glucose, *IGT* impaired glucose tolerance, *T2DM* type 2 diabetes; relative grip strength _max_, maximal of the average of the right or the left grip strength divided by BMI; Relative grip strength _mean_, the mean of the average of both the right and the left grip strength divided by BMI; Relative grip strength _left_, the average of the left grip strength divided by BMI; Relative grip strength _right_, the average of the right grip strength divided by BMI; Absolute grip strength, maximal of the average of the right or the left grip strength

Table 3 shows that, after similar adjustment, in participants with normoglycaemia, fasting glucose was inversely associated with all measures of RGS and AGS in women (*P* for trend from 0.002 to 0.03). In women, the adjusted β (95% CI) was − 0.04 (− 0.08, − 0.005) for RGS _max_, − 0.04 (− 0.07, − 0.02) for RGS _mean_, − 0.05 (− 0.08, − 0.02) for RGS _left_, − 0.04 (− 0.07, − 0.006) for RGS _right_ and − 0.98 (− 1.74, − 0.22) for AGS. However, no association of fasting glucose with grip strength was found in men, when fasting glucose was analyzed as quartiles or continuous (*P* for trend from 0.67 to 0.88). As the associations did not vary by sex (*P* for sex interaction from 0.13 to 0.92), we also conducted analysis in total participants and found consistently inverse associations between fasting glucose and all measures of grip strength (*P* for trend from 0.006 to 0.049). No association between 2hPG and measures of grip strength in normoglycaemic group was found (Supplementary Table 2).

**Table 3:** Grip strength by fasting glucose (in quartiles and as continuous, mmol/l) in 1271 men and 3394 women with normoglycaemia

	Quartile of fasting glucose in normoglycaemia, mmol/l				Adjusted β^†^	*P* for trend
	1st	2nd	3rd	4th		
Men						
Number of subjects	314	310	319	328	–	–
Fasting glucose, mmol/l	4.39 (4.35, 4.42)	4.82 (4.81, 4.83)	5.07 (5.07, 5.08)	5.40 (5.38, 5.42)	–	–
Post-load glucose, mmol/l^†^	5.94 (5.76, 6.13)	6.14 (5.97, 6.30)	6.26 (6.10, 6.43)	6.42 (6.24, 6.60)	0.51 (0.33, 0.68)^***^	< 0.001
Relative grip strength _max_^†^	1.43 (1.37, 1.49)	1.43 (1.37, 1.48)	1.43 (1.37, 1.48)	1.43 (1.37, 1.49)	−0.004 (− 0.06, 0.05)	0.88
Relative grip strength _mean_^†^	1.35 (1.30, 1.41)	1.35 (1.30, 1.40)	1.35 (1.30, 1.39)	1.35 (1.30, 1.40)	−0.01 (− 0.06, 0.04)	0.70
Relative grip strength _left_^†^	1.35 (1.29, 1.41)	1.34 (1.29, 1.40)	1.34 (1.28, 1.40)	1.34 (1.28, 1.40)	−0.01 (− 0.07, 0.05)	0.67
Relative grip strength _right_^†^	1.36 (1.31, 1.41)	1.36 (1.31, 1.40)	1.36 (1.31, 1.40)	1.35 (1.31, 1.40)	−0.008 (− 0.05, 0.04)	0.75
Absolute grip strength, kg^††^	33.27 (31.92, 34.61)	33.21 (32.03, 34.40)	33.18 (32.00, 34.36)	33.14 (31.85, 34.42)	−0.14 (−1.39, 1.12)	0.83
Women						
Number of subjects	840	833	844	877	–	–
Fasting glucose, mmol/l	4.46 (4.45, 4.48)	4.82 (4.82, 4.82)	5.07 (5.07, 5.08)	5.37 (5.37, 5.38)	–	–
Post-load glucose, mmol/l^†^	5.89 (5.82, 5.97)	6.07 (6.01, 6.13)	6.19 (6.13, 6.25)	6.34 (6.26, 6.41)	0.47 (0.37, 0.57)^***^	< 0.001
Relative grip strength _max_^†^	0.95 (0.93, 0.98)	0.94 (0.92, 0.96)	0.93 (0.91, 0.95)	0.92 (0.89, 0.94)	−0.04 (−0.08, −0.005)^*^	0.03
Relative grip strength _mean_^†^	0.90 (0.88, 0.92)	0.89 (0.87, 0.90)	0.87 (0.86, 0.89)	0.86 (0.84, 0.88)	−0.04 (− 0.07, − 0.02)^**^	0.002
Relative grip strength _left_^†^	0.90 (0.87, 0.92)	0.88 (0.86, 0.90)	0.87 (0.85, 0.89)	0.85 (0.83, 0.88)	−0.05 (− 0.08, − 0.02)^**^	0.003
Relative grip strength _right_^†^	0.91 (0.88, 0.93)	0.89 (0.87, 0.91)	0.88 (0.86, 0.90)	0.87 (0.85, 0.89)	−0.04 (− 0.07, − 0.006)^*^	0.02
Absolute grip strength, kg^††^	21.26 (20.70, 21.83)	20.89 (20.45, 21.33)	20.64 (20.19, 21.10)	20.34 (19.77, 20.90)	−0.98 (−1.74, − 0.22)^*^	0.01
Total						
Number of subjects	1154	1143	1163	1205		
Fasting glucose, mmol/l	4.44 (4.43, 4.46)	4.82 (4.816, 4.82)	5.07 (5.07, 5.08)	5.38 (5.37, 5.39)	–	–
Post-load glucose, mmol/l^$^	5.88 (5.82, 5.93)	6.06 (6.03, 6.09)	6.18 (6.15, 6.22)	6.33 (6.28, 6.38)	0.48 (0.40, 0.57) ^***^	< 0.001
Relative grip strength _max_^$^	1.06 (1.04, 1.08)	1.05 (1.04, 1.06)	1.04 (1.03, 1.06)	1.03 (1.02, 1.05)	−0.03 (−0.06, −0.00008)^*^	0.049
Relative grip strength _mean_^$^	1.01 (0.99, 1.02)	0.99 (0.98, 1.00)	0.98 (0.97, 0.99)	0.97 (0.96, 0.99)	−0.04 (− 0.06, − 0.01)^**^	0.006
Relative grip strength _left_^$^	1.00 (0.98, 1.01)	0.98 (0.97, 0.99)	0.97 (0.96, 0.98)	0.96 (0.94, 0.98)	−0.04 (− 0.07, − 0.01)^**^	0.008
Relative grip strength _right_^$^	1.01 (1.00, 1.03)	1.00 (0.99, 1.01)	1.00 (0.99, 1.01)	0.99 (0.97, 1.00)	−0.03 (− 0.06, − 0.003)^*^	0.03
Absolute grip strength, kg^$$^	24.23 (23.82, 24.63)	23.97 (23.72, 24.22)	23.80 (23.54, 24.06)	23.59 (23.21, 23.97)	−0.68 (−1.33, − 0.02)^*^	0.04

Results were shown as mean (95% confidence interval), except for numbers

Relative grip strength _max_, maximal of the average of the right or the left grip strength divided by body mass index (BMI); Relative grip strength _mean_, the mean of the average of both the right and the left grip strength divided by BMI; Relative grip strength _left_, the average of the left grip strength divided by BMI; Relative grip strength _right_, the average of the right grip strength divided by BMI; Absolute grip strength, maximal of the average of the right or the left grip strength

*P* values for sex interaction with fasting glucose in terms of all measures of grip strength were from 0.13 to 0.92

^†^: Adjusted for age, education, smoking status, alcohol use, physical activity, body fat percentage and waist circumference

^††^: Adjusted for age, education, smoking status, alcohol use, physical activity, body fat percentage and waist circumference and BMI

^$^: Adjusted for age, sex, education, smoking status, alcohol use, physical activity, body fat percentage and waist circumference

^$$:^ Adjusted for age, sex, education, smoking status, alcohol use, physical activity, body fat percentage and waist circumference and BMI

*: *P* < 0.05; **: *P* < 0.01; ***: *P* < 0.001

Note: *IFG* impaired fasting glucose, *IGT* impaired glucose tolerance, *T2DM* type 2 diabetes mellitus

**Fig. 2:**
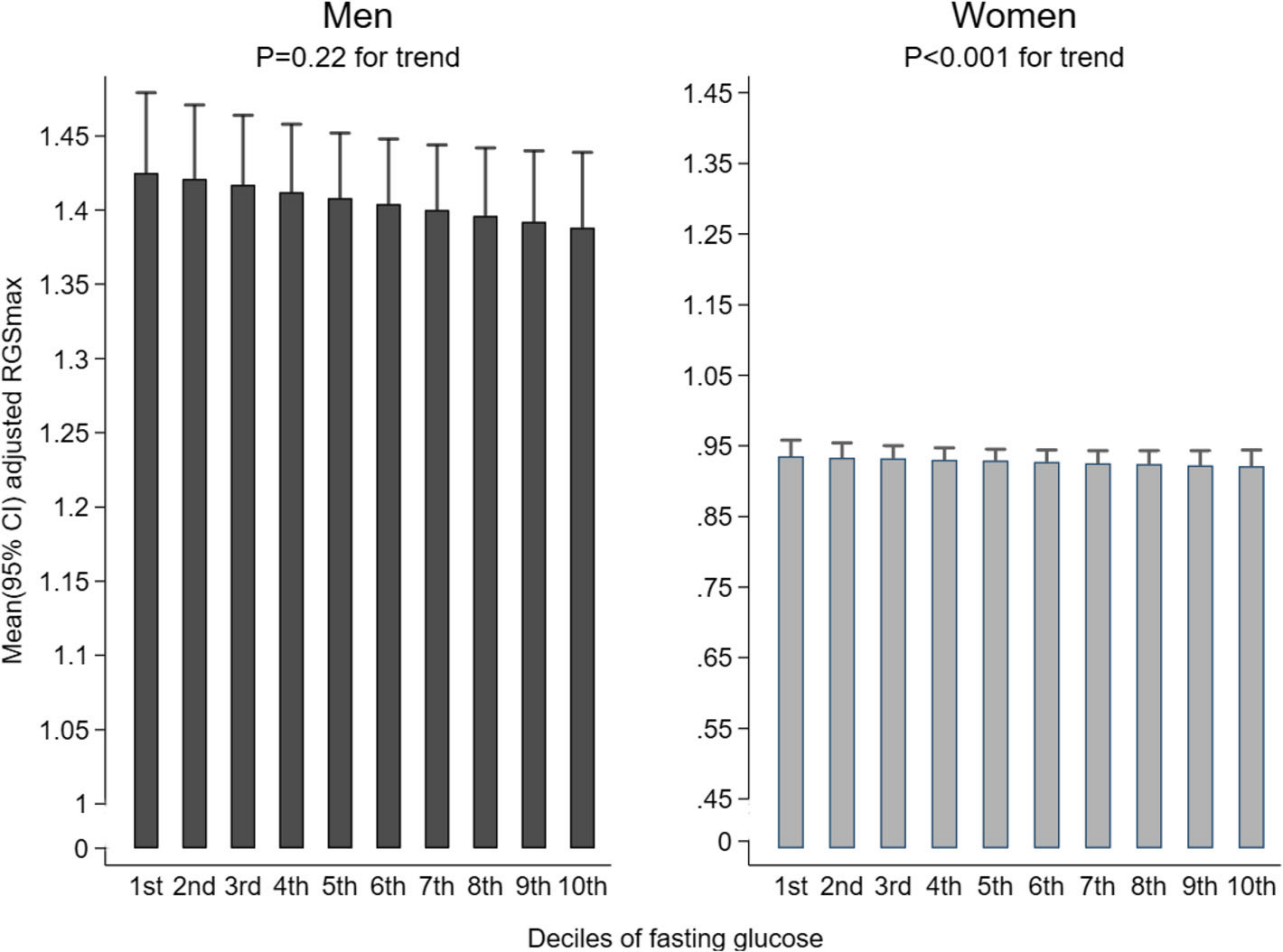
Association between fasting glucose (in deciles and as continuous, mmol/l) and RGS _max_ in participants without T2DM. All the means (95% CIs) were adjusted for age, education, smoking status, alcohol use, physical activity, body fat percentage and waist circumference. Note: r = correlation coefficient

In participants without T2DM, in women, increasing fasting glucose was associated with lower RGS _max_ after full adjustment (*P* < 0.001 for trend), with the RGS _max_ (95% CI) being 0.88 (0.86, 0.90) in the highest decile and 0.95 (0.93, 0.97) in the lowest. In men, there was similar trend between fasting glucose and RGS _max_ but the association was not statistically significant (*P* = 0.22) (Fig. 2). The association of 2hPG with RGS _max_ were not significant in both men and women (*P* value for trend =0.35 and 0.39, respectively) (Supplementary Fig. 2).

## Discussion

To our knowledge, this is the first report showing that in women with normoglycaemia, fasting glucose was inversely associated with grip strength in a dose-response manner. In men, glycemic status was inversely associated with grip strength, with a decreasing grip strength from normoglycaemia to prediabetes then to diabetes, but such association was not statistically significant in women, suggesting the inverse association between fasting glucose and grip strength might have begun below the current diagnostic levels for prediabetes or diabetes in women but is more pronounced in men in later stage. Our results indicate an independent contribution of increasing glycaemia within the normal reference range to weaken grip strength, which may shed light on the mechanical functions of muscle strength.

Although some previous studies showed that lower muscle strength was associated with a higher risk of T2DM [20, 26, 34–36], only three studies reported that higher glucose was associated with lower grip strength [12–14]. Results of both directions should be equally important for mechanistic understanding. Our findings were generally consistent with the three previous studies above [12–14] showing that increasing fasting glucose was inversely associated with grip strength. Of these three studies [12–14], the one on 984 participants in the United State (US) showed that raised hemoglobin A_1c_ was associated with decreased muscle strength in the total participants [14], but whether the association varied by sex was not reported. Another study of 1664 participants in the US showed a significant inverse association of fasting glucose with grip strength in men but not in women, and no association between 2hPG and grip strength in men or women [13]. In the study, they only excluded participants with known diabetes history and those with prediabetes (i.e., IFG and/or IGT) were not excluded from the analysis [13]. However, our study focused on normoglycaemia, which may be the reason for discrepancies between ours and this previous study. In these two studies, in participants without a diagnosis of diabetes, although higher fasting glucose [13] or HbA_1c_ [14] appeared to be associated with lower muscle strength, the results were not statistically significant [13, 14]. The other study on 1391 participants from the United Kingdom found that higher 2hPG and the presence of IGT (defined by elevated 2hPG) was associated with lower grip strength in both men and women but no results on the association of fasting glucose or HbA_1c_ with grip strength were reported [12]. Moreover, the above three studies were not large enough and some important confounders were not adjusted in these three studies, such as smoking status [12, 13], physical activity [12], body fat percentage [12–14] and waist circumference [12–14]. Hence, our results that fasting glucose was inversely associated with all measures of grip strength in women with normoglycaemia with adjustment for a wide set of potential confounding factors with a large sample size should be more robust, and suggest that association between glycemic metabolism and muscle strength could be causal.

Our results showed that higher fasting glucose, but not 2hPG, was a predictor of lower muscle strength, indicating that fasting glucose might be an earlier glycemic indicator and play a more important role than 2hPG in the mechanism impairing physical function. For individuals with IFG, a previous study showed that insulin resistance was more pronounced in women than that in men [37]*.* Women generally had higher body fat percentage and thus may be more prone to insulin resistance [38]. However, in those with normoglycaemia, women were shown to be more insulin sensitive than men [39], which may be another explanation for the differences between genders. Our study also showed that in participants without a diagnosis of diabetes, the inverse association between glycaemia and grip strength was significant in women but not in men. Moreover, our results were also consistent with an experimental study of animals showing that contractile function and force generation were affected by hyperglycemia [40]. Furthermore, as all analyses on grip strength had accounted for BMI, the different associations in men and women were unlikely due to the changes in BMI caused by hyperglycemia. Some explanations for the mechanisms of glucose metabolism and grip strength have been postulated. First, insulin resistance, which is a key underlying metabolic abnormality in hyperglycemia, may induce muscle degradation via the pathways of activation of caspase-3 and the ubiquitin-proteasome proteolytic [41]. Second, elevating fasting glucose is associated with glycogenolysis [42], which may partly contribute to the loss of muscle strength. Third, hyperglycemia may lead to a lower muscle strength through effects on skeletal muscle mitochondria. An impaired bioenergetic capacity dysfunction of muscle mitochondria was found in patients with type 2 diabetes, and higher insulin resistance was associated with more severe damage in mitochondria [43]. Forth, hyperglycemia may lead to a decline in muscle strength via krüppel-like factor 15 protein [44], which has been found to regulate skeletal muscle lipid flux as well as exercise adaptation [45]. Finally, individuals with hyperglycemia were found to have higher levels of inflammatory cytokines such as interleukin-6 and tumor necrosis factor-alpha [46, 47] and these inflammatory factors may also have some adverse effects on muscle mass [48, 49].

The strengths of our study included the large sample size, standardized and comprehensive measurement of anthropometric parameters (body fat percentage, waist circumference and BMI), glycaemia (fasting glucose and 2hPG) and grip strength. However, our study had several limitations. First, as the associations were cross-sectional, whether the association between glycemic status and grip strength is causal is uncertain. Second, there are some factors that may influence the instantaneous strength tests and thus residual confounding could not be completely ruled out, although a wide range of potential confounding factors were adjusted for and the results remained after the adjustment. Third, as all participants were older people who might have relatively lower grip strength, the results may not be applicable to other age groups.

## Conclusions

In conclusion, higher fasting glucose was associated with reduced grip strength in a dose-response manner in men from normoglycaemia to prediabetes then to T2DM, and the association was significant even in women with normoglycaemia. Our findings suggest that lowering glucose across the whole range might be important in preserving muscle strength, especially in aging women. Further studies on the mechanisms and trials are warranted.

## Supplementary information

**Additional file 1.** Supplementary Table 1. Characteristics by quintiles of relative grip strength _max_ in 2498 men and 6638 women in Guangzhou Biobank Cohort Study. SD = standard deviation; Relative grip strength _max_, maximal of the average of the right or the left grip strength divided by body mass index (BMI)

**Additional file 2 **Supplementary Table 2. Grip strength by post-load glucose (in quartiles and as continuous, mmol/l) in 1178 men and 3080 women with normoglycaemia. Results were shown as mean (95% confidence interval), except for numbers. Relative grip strength _max_, maximal of the average of the right or the left grip strength divided by body mass index (BMI); Relative grip strength _mean_, the mean of the average of both the right and the left grip strength divided by BMI; Relative grip strength _left_, the average of the left grip strength divided by BMI; Relative grip strength _right_, the average of the right grip strength divided by BMI; Absolute grip strength, maximal of the average of the right or the left grip strength. ^†^: Adjusted for age, education, smoking status, alcohol use, physical activity, body fat percentage and waist circumference. ^††^: Adjusted for age, education, smoking status, alcohol use, physical activity, body fat percentage and waist circumference and BMI. ^$:^ Adjusted for age, sex, education, smoking status, alcohol use, physical activity, body fat percentage and waist circumference. ^$$^: Adjusted for age, sex, education, smoking status, alcohol use, physical activity, body fat percentage and waist circumference and BMI. ^#^: *P* value for sex interaction with post-load glucose in terms of relative grip strength _mean_ and relative grip strength _right_ was 0.04 and 0.03 respectively. **: *P* < 0.01; ***: *P* < 0.001

**Additional file 3.** Supplementary Figure 1. The matrix diagram of the measures of grip strength

**Additional file 4.** Supplementary Figure 2. Association between 2hPG (in deciles and as continuous, mmol/l) and RGS _max_ in participants without T2DM. All the means (95% CIs) were adjusted for age, education, smoking status, alcohol use, physical activity, body fat percentage and waist circumference

## Data Availability

The datasets used during the current study are available from the corresponding author on reasonable request.

## Abbreviations

RGS: Relative grip strength
DM: Diabetes mellitus
T2DM: Type 2 diabetes mellitus
2hPG: 2-h post-load glucose
GBCS: Guangzhou Biobank Cohort Study
GHHARE: Guangzhou Health and Happiness Association for the Respectable Elders)
BMI: Body mass index
IFG: Impaired fasting glucose
IGT: Impaired glucose tolerance
IFG + IGT: IFG and IGT
AGS: Absolute grip strength
RGS: _max_ Relative grip strength _max_
RGS: _mean_ Relative grip strength _mean_
RGS: _left_ Relative grip strength _left_
RGS: _right_ Relative grip strength _right_
