# Supplementary material for: Glycaemia and hand grip strength in aging people: Guangzhou biobank cohort study
Source: BMC Geriatr. 2020 Oct 12;20:399. doi: 10.1186/s12877-020-01808-0 (PMC7552450; doi:10.1186/s12877-020-01808-0)
Supplement: Supplementary file 1 — Additional file 1. Supplementary Table 1. Characteristics by quintiles of relative grip strength max in 2498 men and 6638 women in Guangzhou Biobank Cohort Study. SD = standard deviation; Relative grip strength max, maximal of the average of the right or the left grip strength divided by body mass index (BMI) [file 12877_2020_1808_MOESM1_ESM.docx]

Supplementary table 1. Characteristics by quintiles of relative grip strength _max_ in 2498 men and 6638 women in Guangzhou Biobank Cohort Study

|  | 1^st^ | 2^nd^ | 3^rd^ | 4^th^ | 5^th^ | P value |
| --- | --- | --- | --- | --- | --- | --- |
| Men | | | | | | |
| Number | 499 | 500 | 499 | 500 | 500 |  |
| Relative grip strength _max_, mean (SD) | 0.86 (0.16) | 1.18 (0.06) | 1.38 (0.06) | 1.59 (0.07) | 1.99 (0.52) | <0.001 |
| Absolute grip strength, mean (SD) | 21.78 (5.01) | 28.40 (3.88) | 32.70 (4.28) | 36.42 (4.66) | 43.20 (11.52) | <0.001 |
| Age, years, mean (SD) | 71.41 (6.86) | 68.53 (6.43) | 67.95 (5.97) | 67.12 (6.28) | 65.21 (5.76) | <0.001 |
| Education, % |  |  |  |  |  |  |
| Primary or below | 29.86 | 27.00 | 25.25 | 27.66 | 26.40 | 0.37 |
| Middle school | 52.91 | 53.40 | 59.92 | 56.31 | 56.40 |  |
| College or above | 17.23 | 19.60 | 14.83 | 16.03 | 17.20 |  |
| Smoking status, % |  |  |  |  |  |  |
| Never | 46.79 | 44.69 | 46.39 | 41.97 | 40.20 | <0.001 |
| Former | 32.53 | 26.25 | 26.10 | 29.92 | 22.80 |  |
| Current | 20.68 | 29.06 | 27.51 | 28.11 | 37.00 |  |
| Alcohol use, % |  |  |  |  |  |  |
| Never | 46.89 | 35.00 | 31.26 | 23.60 | 20.60 | <0.001 |
| Former | 0.60 | 0.20 | 0.60 | 1.40 | 1.00 |  |
| Current | 52.51 | 64.80 | 68.14 | 75.00 | 78.40 |  |
| Physical activity, % |  |  |  |  |  |  |
| Inactive | 1.20 | 1.80 | 1.40 | 1.80 | 3.20 | 0.03 |
| Minimally active | 26.85 | 23.40 | 28.46 | 31.80 | 29.40 |  |
| Active | 71.94 | 74.80 | 70.14 | 66.40 | 67.40 |  |
| Poor health, % | 61.05 | 58.94 | 60.84 | 61.49 | 58.95 | 0.87 |
| Body fat, %, median (SD) | 24.28 (5.99) | 23.06 (5.35) | 22.37 (5.72) | 21.35 (6.25) | 19.80 (5.86) | <0.001 |
| Waist circumference, cm, mean (SD) | 88.31 (8.98) | 85.98 (9.13) | 84.51 (8.74) | 83.15 (8.38) | 80.42 (8.22) | <0.001 |
| Women | | | | | | |
| Number | 1327 | 1328 | 1327 | 1328 | 1328 |  |
| Relative grip strength _max_, mean (SD) | 0.45 (0.13) | 0.70 (0.05) | 0.85 (0.04) | 1.01 (0.05) | 1.30 (0.43) | <0.001 |
| Absolute grip strength, mean (SD) | 11.34 (3.72) | 17.27 (2.74) | 20.74 (2.84) | 23.71 (3.05) | 28.36 (9.26) | <0.001 |
| Age, years, mean (SD) | 66.67 (7.65) | 64.38 (6.90) | 64.60 (6.78) | 64.74 (6.52) | 63.31 (5.81) | <0.001 |
| Education, % |  |  |  |  |  |  |
| Primary or below | 44.99 | 41.49 | 43.93 | 44.88 | 42.39 | 0.18 |
| Middle school | 47.48 | 51.58 | 49.51 | 47.97 | 51.96 |  |
| College or above | 7.54 | 6.93 | 6.56 | 7.15 | 5.65 |  |
| Smoking status, % |  |  |  |  |  |  |
| Never | 97.89 | 97.73 | 96.66 | 97.49 | 98.10 | 0.44 |
| Former | 0.98 | 1.06 | 1.59 | 1.22 | 0.68 |  |
| Current | 1.13 | 1.21 | 1.75 | 1.29 | 1.21 |  |
| Alcohol use, % |  |  |  |  |  |  |
| Never | 59.13 | 54.15 | 46.87 | 42.76 | 37.36 | <0.001 |
| Former | 0.15 | 0.08 | 0.38 | 0.38 | 0.38 |  |
| Current | 40.72 | 45.78 | 52.75 | 56.86 | 62.26 |  |
| Physical activity, % |  |  |  |  |  |  |
| Inactive | 1.36 | 1.73 | 3.39 | 3.24 | 2.84 | <0.001 |
| Minimally active | 19.14 | 21.31 | 24.49 | 23.64 | 24.32 |  |
| Active | 79.50 | 76.96 | 72.12 | 73.12 | 72.82 |  |
| Poor health, % | 62.15 | 64.60 | 66.36 | 65.81 | 66.36 | 0.12 |
| Body fat, %, mean (SD) | 35.06 (7.30) | 34.44 (7.00) | 33.57 (6.33) | 32.04 (6.03) | 29.38 (6.29) | <0.001 |
| Waist circumference, cm, mean (SD) | 83.48 (9.10) | 82.91 (9.33) | 82.58 (8.70) | 80.89 (8.06) | 78.33 (8.19) | <0.001 |

SD = standard deviation; Relative grip strength _max_, maximal of the average of the right or the left grip strength divided by body mass index (BMI).
