# Supplementary material for: Glycaemia and hand grip strength in aging people: Guangzhou biobank cohort study
Source: BMC Geriatr. 2020 Oct 12;20:399. doi: 10.1186/s12877-020-01808-0 (PMC7552450; doi:10.1186/s12877-020-01808-0)
Supplement: Supplementary file 2 — Additional file 2 Supplementary Table 2. Grip strength by post-load glucose (in quartiles and as continuous, mmol/l) in 1178 men and 3080 women with normoglycaemia. Results were shown as mean (95% confidence interval), except for numbers. Relative grip strength max, maximal of the average of the right or the left grip strength divided by body mass index (BMI); Relative grip strength mean, the mean of the average of both the right and the left grip strength divided by BMI; Relative grip strength left, the average of the left grip strength divided by BMI; Relative grip strength right, the average of the right grip strength divided by BMI; Absolute grip strength, maximal of the average of the right or the left grip strength. †: Adjusted for age, education, smoking status, alcohol use, physical activity, body fat percentage and waist circumference. ††: Adjusted for age, education, smoking status, alcohol use, physical activity, body fat percentage and waist circumference and BMI. $: Adjusted for age, sex, education, smoking status, alcohol use, physical activity, body fat percentage and waist circumference. $$: Adjusted for age, sex, education, smoking status, alcohol use, physical activity, body fat percentage and waist circumference and BMI. #: P value for sex interaction with post-load glucose in terms of relative grip strength mean and relative grip strength right was 0.04 and 0.03 respectively. **: P < 0.01; ***: P < 0.001 [file 12877_2020_1808_MOESM2_ESM.docx]

Supplementary table 2. Grip strength by post-load glucose (in quartiles and as continuous, mmol/l) in 1178 men and 3080 women with normoglycaemia

|  | Quartile of post-load glucose in normoglycaemia, mmol/l | | | | Adjusted β^†^ | P for trend |
| --- | --- | --- | --- | --- | --- | --- |
|  | 1^st^ | 2^nd^ | 3^rd^ | 4^th^ |  |  |
| Men | | | | | | |
| Number of subjects | 294 | 293 | 295 | 296 | - | - |
| Post-load glucose, mmol/l | 4.39 (4.30, 4.48) | 5.72 (5.70, 5.75) | 6.51 (6.48, 6.53) | 7.46 (7.37, 7.54) | - | - |
| Fasting glucose, mmol/l^†^ | 4.87 (4.81, 4.93) | 4.93 (4.88, 4.98) | 4.97 (4.92, 5.02) | 5.01 (4.96, 5.07) | 0.05 (0.04, 0.07)^***^ | <0.001 |
| Relative grip strength _max_^†^ | 1.42 (1.36, 1.48) | 1.42 (1.36, 1.47) | 1.42 (1.36, 1.47) | 1.41 (1.35, 1.47) | -0.002 (-0.02, 0.02) | 0.83 |
| Relative grip strength _mean_^†#^ | 1.35 (1.30, 1.40) | 1.34 (1.30, 1.39) | 1.34 (1.29, 1.39) | 1.34 (1.29, 1.39) | -0.005 (-0.02, 0.01) | 0.53 |
| Relative grip strength _left_^†^ | 1.34 (1.28, 1.40) | 1.33 (1.28, 1.39) | 1.33 (1.27, 1.39) | 1.33 (1.26, 1.39) | -0.005 (-0.02, 0.01) | 0.61 |
| Relative grip strength _right_^†#^ | 1.36 (1.31, 1.42) | 1.36 (1.31, 1.40) | 1.35 (1.31, 1.40) | 1.35 (1.30, 1.40) | -0.006 (-0.02, 0.01) | 0.44 |
| Absolute grip strength, kg^††^ | 32.81 (31.50, 34.12) | 32.90 (31.74, 34.06) | 32.95 (31.79, 34.11) | 33.01 (31.77, 34.26) | 0.08 (-0.33, 0.48) | 0.72 |
| Women | | | | | | |
| Number of subjects | 765 | 775 | 766 | 774 | - | - |
| Post-load glucose, mmol/l | 4.78 (4.73, 4.82) | 5.87 (5.86, 5.89) | 6.57 (6.55, 6.58) | 7.35 (7.32, 7.38) | - | - |
| Fasting glucose, mmol/l^†^ | 4.82 (4.79, 4.84) | 4.88 (4.86, 4.91) | 4.93 (4.91, 4.95) | 4.98 (4.95, 5.00) | 0.06 (0.05, 0.07)^***^ | <0.001 |
| Relative grip strength _max_^†^ | 0.94 (0.91, 0.97) | 0.95 (0.92, 0.97) | 0.95 (0.93, 0.97) | 0.96 (0.93, 0.98) | 0.006 (-0.008, 0.02) | 0.39 |
| Relative grip strength _mean_^†#^ | 0.89 (0.86, 0.91) | 0.89 (0.88, 0.91) | 0.90 (0.88, 0.92) | 0.90 (0.88, 0.92) | 0.005 (-0.006, 0.02) | 0.35 |
| Relative grip strength _left_^†^ | 0.88 (0.85, 0.91) | 0.89 (0.87, 0.91) | 0.89 (0.87, 0.91) | 0.90 (0.87, 0.92) | 0.006 (-0.007, 0.02) | 0.38 |
| Relative grip strength _right_^†#^ | 0.89 (0.87, 0.92) | 0.90 (0.88, 0.92) | 0.90 (0.88, 0.92) | 0.91 (0.88, 0.93) | 0.006 (-0.006, 0.02) | 0.32 |
| Absolute grip strength, kg^††^ | 20.81 (20.17, 21.45) | 21.05 (20.55, 21.54) | 21.19 (20.69, 21.69) | 21.36 (20.76, 21.96) | 0.20 (-0.08, 0.49) | 0.17 |
| Total | | | | | | |
| Number of subjects | 1059 | 1068 | 1061 | 1070 |  |  |
| Post-load glucose, mmol/l | 4.67 (4.63, 4.71) | 5.83 (5.82, 5.85) | 6.55 (6.54, 6.56) | 7.38 (7.35, 7.41) | - | - |
| Fasting glucose, mmol/l^$^ | 4.84 (4.82, 4.86) | 4.91 (4.90, 4.92) | 4.95 (4.94, 4.96) | 5.00 (4.98, 5.01) | 0.06 (0.05, 0.07) ^***^ | <0.001 |
| Relative grip strength _max_^$^ | 1.05 (1.03, 1.07) | 1.06 (1.04, 1.07) | 1.06 (1.05, 1.07) | 1.06 (1.04, 1.08) | 0.003 (-0.008, 0.01) | 0.54 |
| Relative grip strength _mean_^$#^ | 0.99 (0.98, 1.01) | 1.00 (0.99, 1.01) | 1.00 (0.99, 1.01) | 1.00 (0.98, 1.01) | 0.002 (-0.007, 0.01) | 0.72 |
| Relative grip strength _left_^†^ | 0.98 (0.97, 1.00) | 0.99 (0.98, 1.00) | 0.99 (0.98, 1.00) | 0.99 (0.97, 1.01) | 0.002 (-0.008, 0.01) | 0.69 |
| Relative grip strength _right_^$#^ | 1.01 (0.99, 1.02) | 1.01 (1.00, 1.02) | 1.01 (1.00, 1.02) | 1.01 (0.99, 1.03) | 0.002 (-0.008, 0.01) | 0.72 |
| Absolute grip strength, kg^$$^ | 23.89 (23.47, 24.32) | 24.09 (23.82, 24.35) | 24.20 (23.93, 24.48) | 24.34 (23.95, 24.73) | 0.17 (-0.07, 0.40) | 0.17 |

Results were shown as mean (95% confidence interval), except for numbers.

Relative grip strength _max_, maximal of the average of the right or the left grip strength divided by body mass index (BMI); Relative grip strength _mean_, the mean of the average of both the right and the left grip strength divided by BMI; Relative grip strength _left_, the average of the left grip strength divided by BMI; Relative grip strength _right_, the average of the right grip strength divided by BMI; Absolute grip strength, maximal of the average of the right or the left grip strength.

^†^: Adjusted for age, education, smoking status, alcohol use, physical activity, body fat percentage and waist circumference.

^††^: Adjusted for age, education, smoking status, alcohol use, physical activity, body fat percentage and waist circumference and BMI.

^$:^ Adjusted for age, sex, education, smoking status, alcohol use, physical activity, body fat percentage and waist circumference.

^$$^: Adjusted for age, sex, education, smoking status, alcohol use, physical activity, body fat percentage and waist circumference and BMI.

^#^: P value for sex interaction with post-load glucose in terms of relative grip strength _mean_ and relative grip strength _right_ was 0.04 and 0.03 respectively.

**: P<0.01; ***: P<0.001.
