# Supplementary figures and images for: Glycaemia and hand grip strength in aging people: Guangzhou biobank cohort study
Source: BMC Geriatr. 2020 Oct 12;20:399. doi: 10.1186/s12877-020-01808-0 (PMC7552450; doi:10.1186/s12877-020-01808-0)

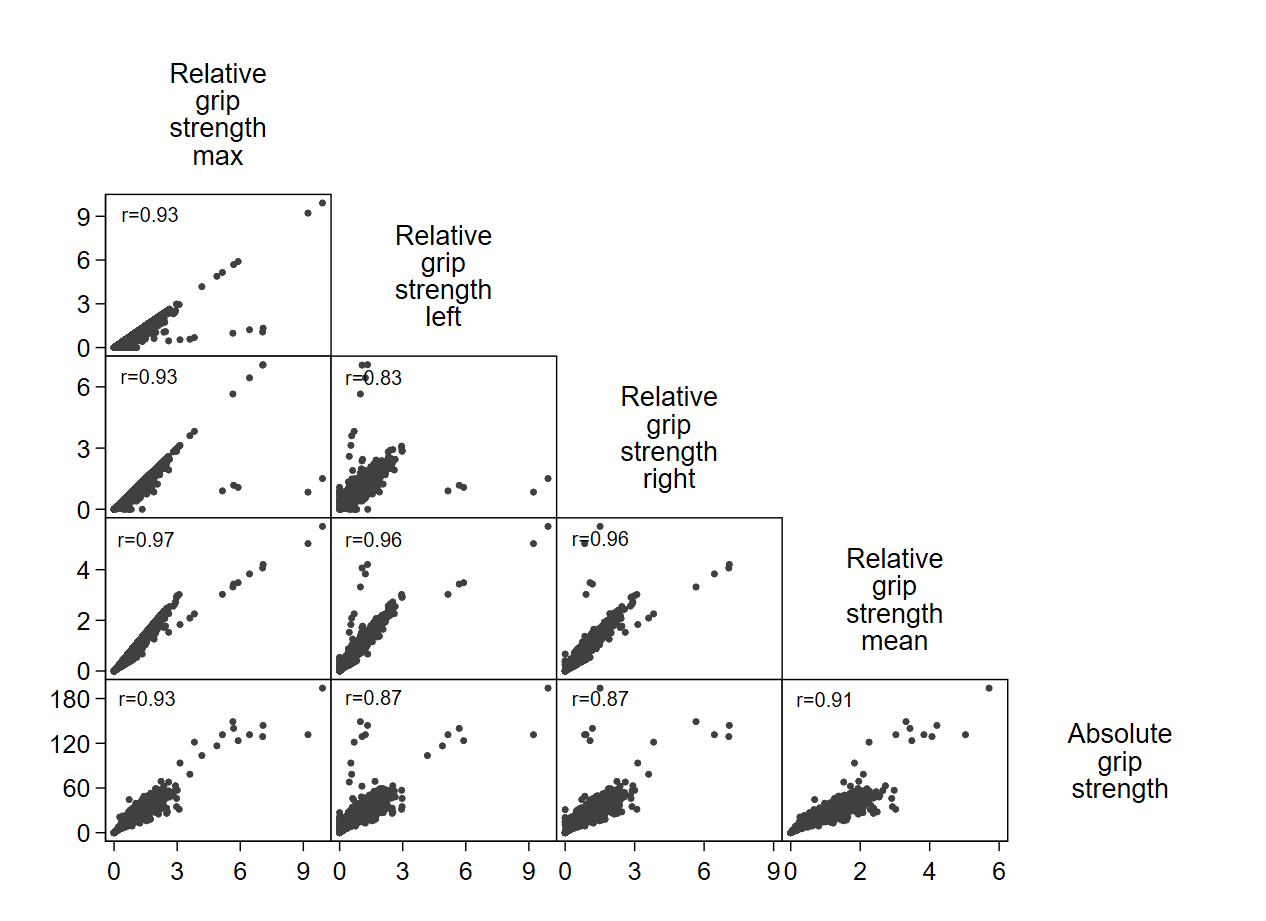

Supplement: Supplementary file 3 — Additional file 3. Supplementary Figure 1. The matrix diagram of the measures of grip strength [file 12877_2020_1808_MOESM3_ESM.tif]

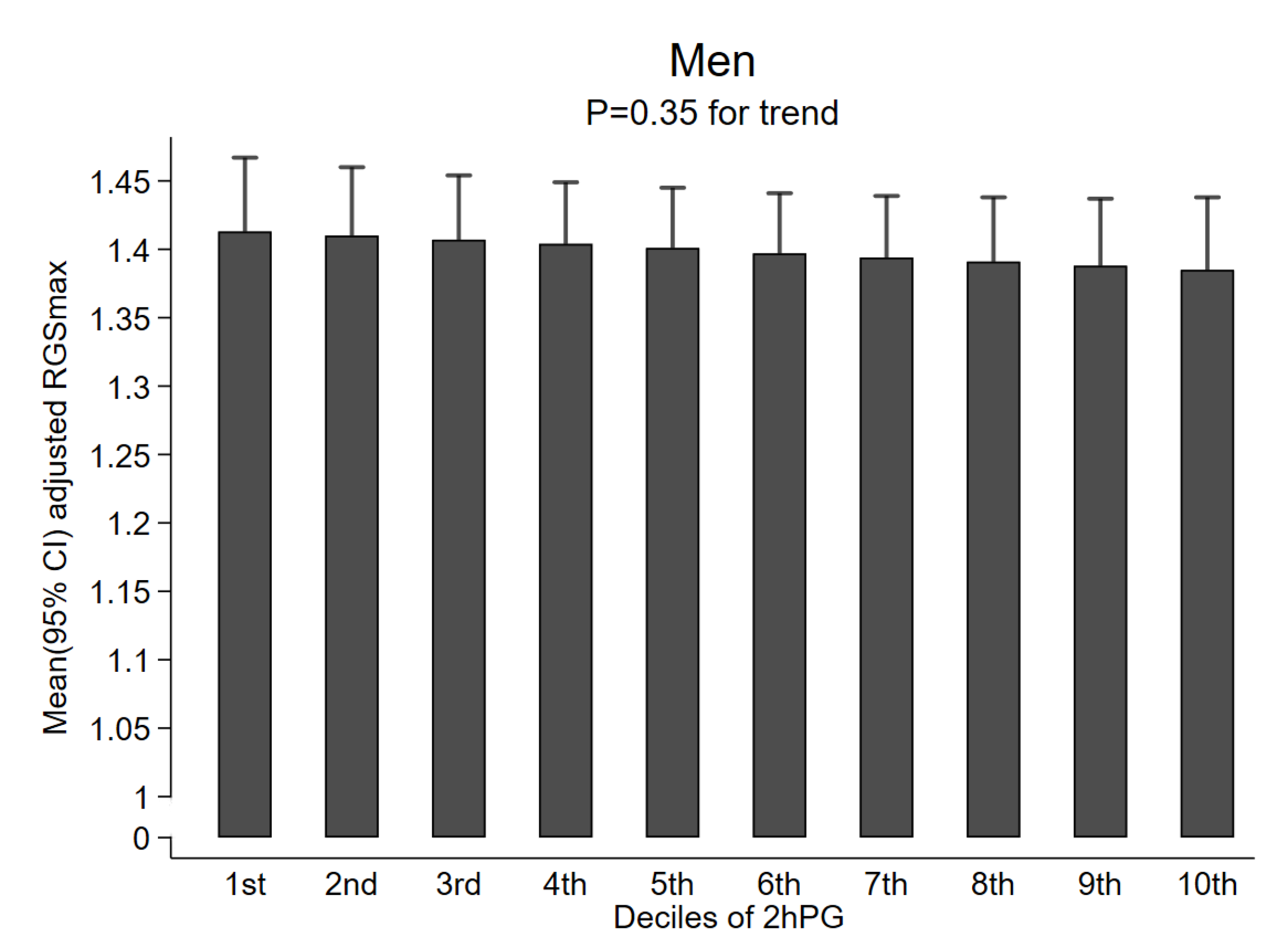

Supplement: Supplementary file 4 — Additional file 4. Supplementary Figure 2. Association between 2hPG (in deciles and as continuous, mmol/l) and RGS max in participants without T2DM. All the means (95% CIs) were adjusted for age, education, smoking status, alcohol use, physical activity, body fat percentage and waist circumference [file 12877_2020_1808_MOESM4_ESM.zip › Supplementary figure_2_menR2.tif]

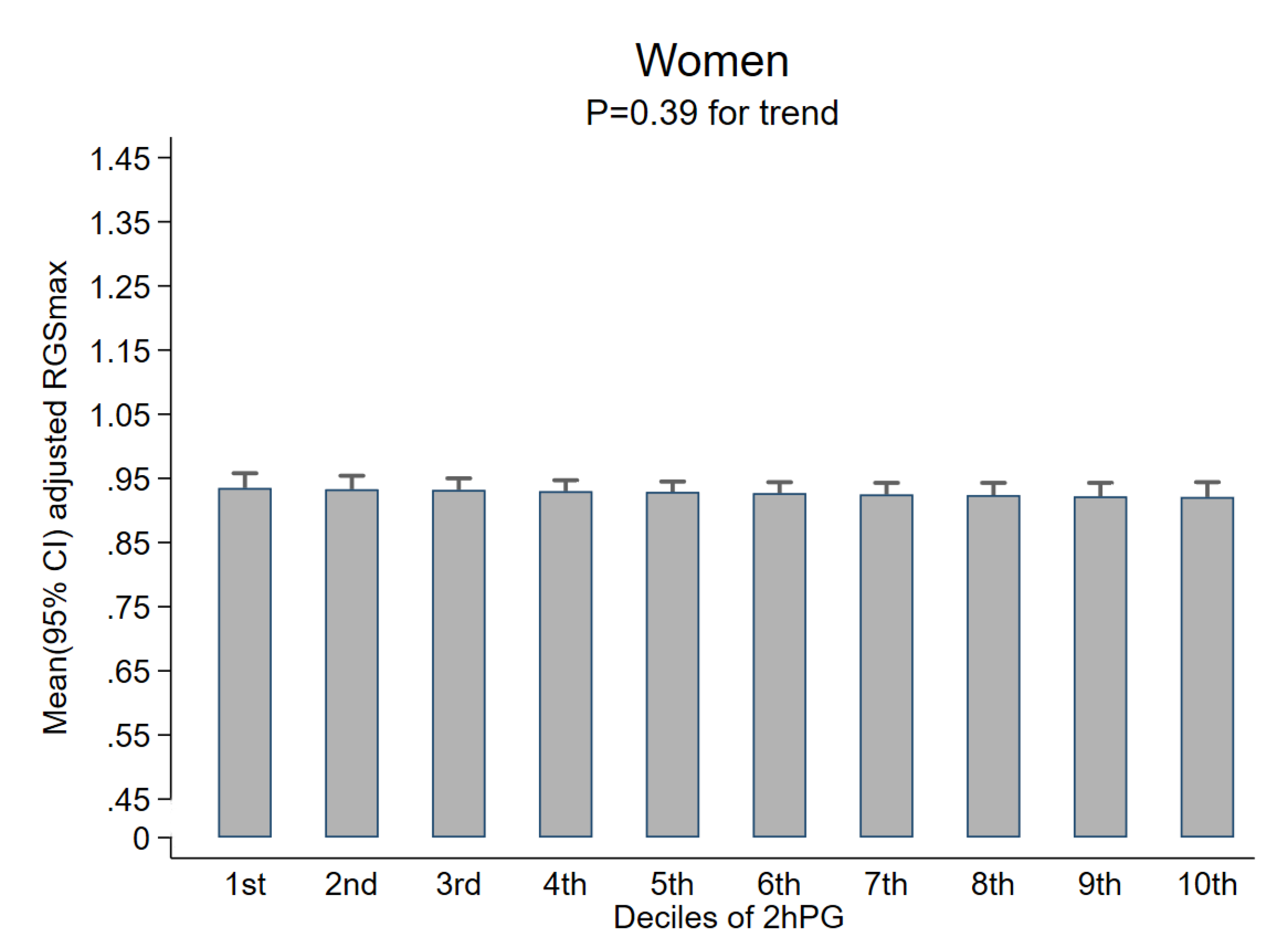

Supplement: Supplementary file 4 — Additional file 4. Supplementary Figure 2. Association between 2hPG (in deciles and as continuous, mmol/l) and RGS max in participants without T2DM. All the means (95% CIs) were adjusted for age, education, smoking status, alcohol use, physical activity, body fat percentage and waist circumference [file 12877_2020_1808_MOESM4_ESM.zip › Supplementary figure_2_womenR2.tif]

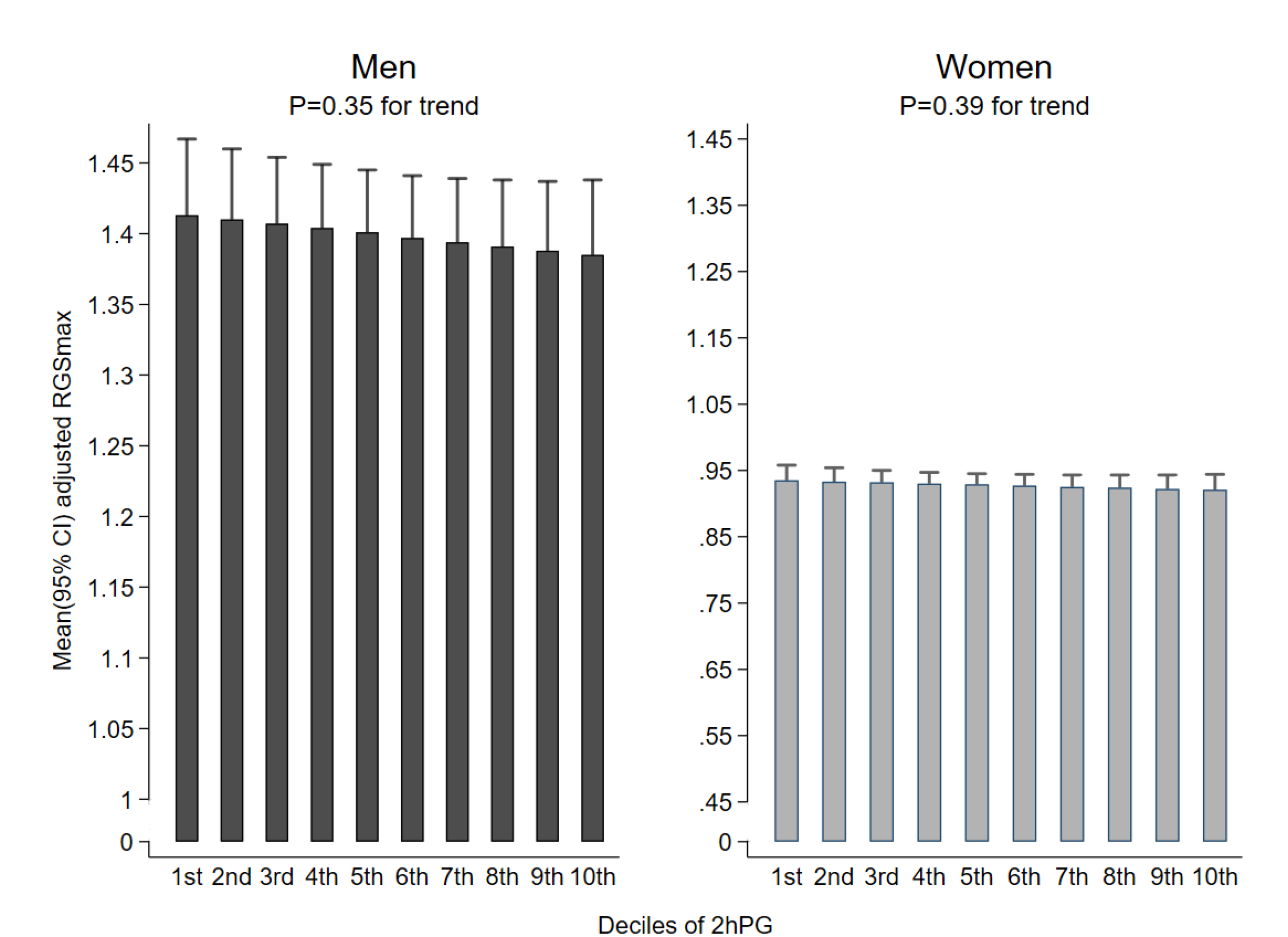

Supplement: Supplementary file 4 — Additional file 4. Supplementary Figure 2. Association between 2hPG (in deciles and as continuous, mmol/l) and RGS max in participants without T2DM. All the means (95% CIs) were adjusted for age, education, smoking status, alcohol use, physical activity, body fat percentage and waist circumference [file 12877_2020_1808_MOESM4_ESM.zip › Supplementary figureR2_2.tif]
